# Expression, biochemical and structural characterization of high-specific-activity β-amylase from *Bacillus aryabhattai* GEL-09 for application in starch hydrolysis

**DOI:** 10.1186/s12934-021-01649-5

**Published:** 2021-09-18

**Authors:** Xuguo Duan, Qiuyu Zhu, Xinyi Zhang, Zhenyan Shen, Yue Huang

**Affiliations:** College of Light Industry and Food Engineering, Nanjing Forestry University, Nanjing, 210037 Jiangsu China

**Keywords:** β-amylase, *Bacillus aryabhattai*, Recombinant expression, Structural properties

## Abstract

**Background:**

β-amylase (EC 3.2.1.2) is an exo-enzyme that shows high specificity for cleaving the α-1,4-glucosidic linkage of starch from the non-reducing end, thereby liberating maltose. In this study, we heterologously expressed and characterized a novel β-amylase from *Bacillus aryabhattai*.

**Results:**

The amino acid-sequence alignment showed that the enzyme shared the highest sequence identity with β-amylase from *Bacillus flexus* (80.73%) followed by *Bacillus cereus* (71.38%). Structural comparison revealed the existence of an additional starch-binding domain (SBD) at the C-terminus of *B. aryabhattai* β-amylase, which is notably different from plant β-amylases. The recombinant enzyme purified 4.7-fold to homogeneity, with a molecular weight of ~ 57.6 kDa and maximal activity at pH 6.5 and 50 °C. Notably, the enzyme exhibited the highest specific activity (3798.9 U/mg) among reported mesothermal microbial β-amylases and the highest specificity for soluble starch, followed by corn starch. Kinetic analysis showed that the *K*_m_ and *k*_cat_ values were 9.9 mg/mL and 116961.1 s^− 1^, respectively. The optimal reaction conditions to produce maltose from starch resulted in a maximal yield of 87.0%. Moreover, molecular docking suggested that *B. aryabhattai* β-amylase could efficiently recognize and hydrolyze maltotetraose substrate.

**Conclusions:**

These results suggested that *B. aryabhattai* β-amylase could be a potential candidate for use in the industrial production of maltose from starch.

**Supplementary Information:**

The online version contains supplementary material available at 10.1186/s12934-021-01649-5.

## Background

Starch is well known for its easy availability, renewability, and low cost [1, 2], as well as its versatility as a biomaterial used in foods, textiles, pharmaceuticals, and adhesives, and as starting material for alcohol-based fuels [3]. Commercially available starches are obtained from various sources, including wheat and corn (cereals), potato (tubers), and cassava (root) [4]. The processing of starch can produce glucose, maltose, fructose syrup, maltodextrin, ethanol, organic acid, and antibiotics, with amylolytic enzymes, including α-amylase, β-amylase, pullulanase, and glucoamylase, widely used in starch processing. β-amylase (EC 3.2.1.2) is an exo-enzyme that shows high specificity for cleaving the α-1,4-glucosidic linkage of starch from the non-reducing end, thereby liberating maltose [5, 6]. β-amylase is a member of family 14 of glycoside hydrolases [7] and widely used in starch processing primarily for producing maltose syrup and brewing [8]. Recently, the increasing industrial application of β-amylase has elevated the attention given to its production [9, 10].

β-amylase plays an important role in organisms and is widely distributed in nature, specifically in microorganisms and plants [11]. In plants, β-amylase is strongly associated with fruit development, ripening, seed germination, and abiotic stress response. Plants, such as sweet potato, soybean, and barley, are frequently used as resources of β-amylase production in industrial fields [6, 12]. However, plant-sourced β-amylases have drawbacks, including their requirement for large amounts of grains, complex preparation processes, low storage stability, and high production cost, which restrict their further application. Moreover, grain consumption has continued to rise over the previous 20 years, resulting in concerns regarding stable supplies of plant-based enzymes [13]. Therefore, it is urgent to search for new β-amylase resources.

Microorganisms are another alternative for β-amylase acquisition. *Bacillus megaterium* β-amylase was first isolated and characterized in 1974 [14], with several species of microorganisms having been subsequently identified as harboring β-amylase-producing capacity, and numerous microbial β-amylases having been characterized in detail [15, 16]. β-amylase-producing microorganisms mainly include Gram-positive bacteria [13, 17, 18], halophiles [16, 19], and thermophiles [20]. Microbial β-amylase is structurally similar to plant β-amylase (~ 20–40%); however, compared with plant β-amylase, microbial β-amylase has numerous advantages. First, the production process is unaffected by season and climate, the downstream processes is simple, and the product is uniform in nature and more stable [10]. Additionally, bacterial β-amylase can digest raw starch [21], which increases its potential applications in starch-processing areas. Moreover, it is easier to modify microbial β-amylase in order to adapt it to flexible and diverse application requirements. Rational and irrational molecular modifications have been used to increase the optimal pH and enhance the catalytic activity of β-amylases [18, 21, 22].

However, due to their low enzyme activity and high production costs, there is limited production of β-amylases from wild-type microorganisms, especially extreme microorganisms. Although mutagenic breeding [23] and process optimization^*17*^ have been applied to enhance enzyme productivity, there remain challenges for industrial applications. β-Amylase-encoding genes have been cloned and heterogeneously expressed in different host cells in order to increase production [24], and recombinant DNA technology has improved protein yields and helped produce commercial enzymes that were previously unmanufacturable. Notably, the fermentation activity (U/mL) of recombinant β-amylase is mainly affected by two factors: protein yield (mg/mL) and enzyme specific activity (U/mg). However, previous studies report that microbial β-amylase exhibits low specific activity [18, 25], which dramatically influences fermentation activity (U/mL). Therefore, identifying a β-amylase that can be efficiently produced in a heterologous expression system and exhibiting high specific activity is critical.

In our previous study, we screened a wild-type β-amylase-producing strain of *Bacillus* sp. GEL-09 from the shallow soil of a cassava field in Guanxi China in order to obtain a amylolytic enzyme more suitable for starch processing. This strain was identified as *Bacillus aryabhatta* (CCTCC M2017320) and deposited in the China Center for Type Culture Collection (CCTCC). In the present study, we cloned and expressed the gene encoding β-amylase from *B. aryabhattai* CCTCC M2017320 and characterized the recombinant enzyme. Furthermore, we applied this β-amylase for starch hydrolysis to produce maltose.

## Results and discussion

### Cloning of *AmyBa*

In our previous study, we screened the β-amylase-producing strain *B. aryabhattai* (CCTCC M2017320) from shallow soil of a cassava field; however, detailed information regarding the coding sequence and properties of the enzyme remained unknown. Recently, genome-assembly and annotation information for *B. aryabhattai* were determined and deposited into GenBank. After searching for putative β-amylases against the *B. aryabhattai* genome, we identified a hypothetical protein annotated as β-amylase (WP_033580731.1), with sequence alignment showing that the protein shared highest similarity (80.73%) with that of a well-characterized β-amylase from *Bacillus flexus*.

**Fig. 1:**
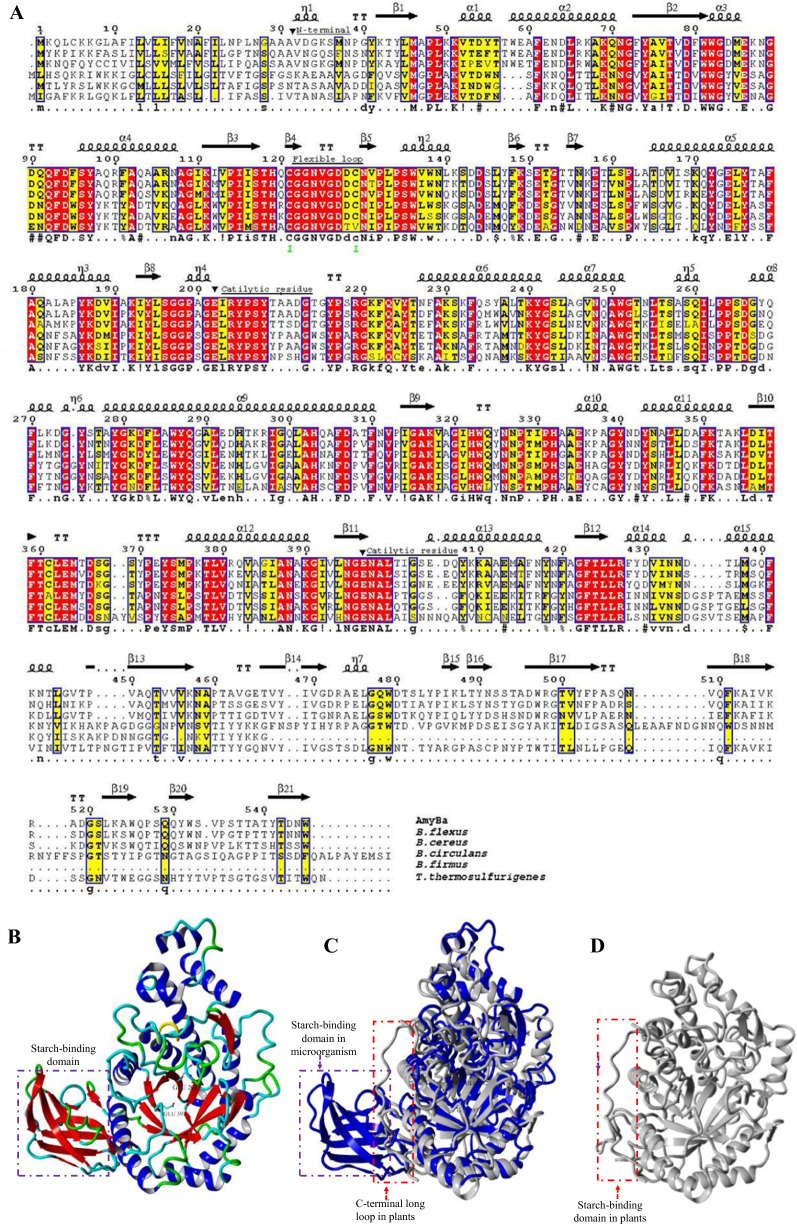
Sequence and structure analysis of AmyBa. **A** Multiple sequence alignment of β-amylases. The strictly conserved residues are shown on a red background, and the highly conserved residues shown on a yellow background. The secondary structure elements are shown for *B. cereus* β-amylase (PDB ID: 5BCA). The signal-peptide-cleavage site and two catalytic residues (E) are indicated by black triangles (black inverted triangle). Conservation of the flexible loop motif (HXCGGNVGD) is noted. β-amylase accession numbers are as follows: *B. aryabhattai* (WP_033580731.1), *B. cereus* (P36924.2), *B. flexus* (RIV10038.1), *B. firmus* (P96513.1), *B. circulans* (P06547.1), *T. thermosulfurigenes* (P19584.1). **B** Three-dimensional molecular model of *B. aryabhattai* β-amylase (AmyBa). **C** Superimposition of AmyBa (Blue) and soybean β-amylases (PDB ID: 1Q6C) (gray) and **D** (PDB ID: 1Q6C) (gray). The C-terminal SBD in microbial β-amylases (box, purple) and the C-terminal loop in plants (box, red)

A pair of primers were then synthesized, and the β-amylase-encoding gene (*AmyBa*) was amplified by PCR using the genomic DNA of *B. aryabhattai* CCTCC M2017320 as a template. DNA sequencing showed a length of *AmyBa* of 1635 bp, encoding 545 aa, including a 31-aa signal peptide (Fig. 1A) and a 514-aa mature protein. The mature protein contains a catalytic domain (Glyco_hydro_14; glycosyl hydrolase family 14) and a C-terminal carbohydrate-binding domain (CBM20). The molecular weight and pI value of mature AmyBa were estimated at 56.86 kDa and 6.6, respectively.

### Sequence and structure analysis of AmyBa

**Fig. 2:**
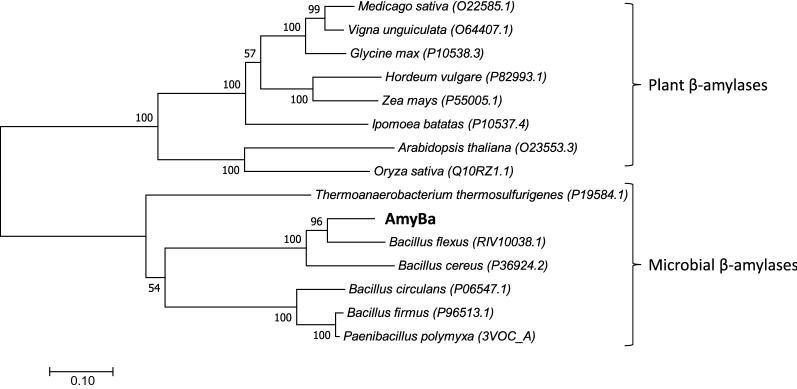
Phylogenetic tree of β-amylases identified from different microorganisms and plants. The phylogenetic tree was created by the neighbor-joining method using MEGA 7.0. The evolutionary distance and branch length are shown. The scale bar corresponds to a genetic distance of 0.1 substitution per position

Numerous β-amylases have been identified, most of which are from plants, whereas only seven microbial β-amylases have been sequenced and characterized. Here, we performed multiple sequence alignments and cladogram analyses to investigate the evolutionary relationships among β-amylases. The results clearly showed two major clusters: one for microorganisms and another for plants (Fig. 2). The two distinct clusters within the tree suggested the presence of two evolutionarily diversified clades (Fig. 2; Table 1), revealing that AmyBa shares < 30% identity with all plant β-amylases, whereas it shares > 45% identity with other microbial β-amylases. Additionally, AmyBa shares the highest identity with the β-amylase from *B. flexus* (80.73%) followed by *B. cereus* (71.38%) [7, 21], *Paenibacillus polymyxa* (50.90%), *Bacillus firmus* (49.78%), *Bacillus circulans* (46.31%) [18], and *Thermoanaerobacterium thermosulfurigenes* (45.06%) [20].

**Table 1:** A pairwise comparison between the amino acid sequences of the β-amylases

β-amylase sequence from	Similarity value (%) for β-amylase sequence from								
	*Barley*	*Sweet potato*	*Soybean*	*B.cereus*	*B.aryabhattai*	*B.flexus*	*T.thermosulfurigenes*	*B.circulans*	*B.firmus*
*Barley*	100.0	60.	67.1	28.4	29.5	29.1	34.3	31.1	33.8
*Sweet*		100.0	68.3	28.0	29.3	29.1	32.5	29.9	32.3
*Soybean*			100.0	27.9	28.4	28.6	32.9	31.6	32.8
*B.cereus*				100.0	71.4	69.4	43.1	43.0	48.5
*B.aryabhattai*					100.0	80.7	45.1	46.3	49.8
*B.flexus*						100.0	46.9	45.9	50.0
*T.thermosulfurigenes*							100.0	51.2	53.8
*B.circulans*								100.0	82.3
*B.firmus*									100.0

The sequences are from the following sources: *B. aryabhattai* (This study), *B. cereus* (P36924.2), *B. flexus* (RIV10038.1), *B. firmus* (P96513.1), *B. circulans* (P06547.1), *T. thermosulfurigenes* (P19584.1), *Sweet potato* (P10537.4), *Soybean* (P10538.3), *Barley* (P82993.1)

Plant and microbial β-amylases are similar in terms of the catalytic roles of active site residues and the three-dimensional structures; however, microbial β-amylase can digest raw starch, whereas plant β-amylase cannot [7, 11, 26–28]. Investigation of the multiple sequence alignment of β-amylases from microorganisms and plants indicated strict conservation of most residues located in the active sites, with conservation of a flexible loop motif (HXCGGNVGD) and the catalytic residues Glu202 and Glu 397 in AmyBa observed across species (Fig. 1A). Crystal structures of two microbial β-amylases (from *B. cereus* and *P. polymyxa*) and four plant β-amylases (from barley, sweet potato, soybean, and wheat) have been determined and deposited in the PDB [PDB IDs: 5BCA [29], 2XFR, 5WQS [11], 1Q6C [27], and 6GER, respectively]. Both classes of β-amylases are characterized by a canonical (β/α)_8_ barrel that comprise the active site. Thirty predictive models of the AmyBa were generated based on the homologues structure of β-amylase from *B. cereus* (PDB ID: 1J10), and model quality and validation were performed using PROCHECK [30], Verify3D [31] and ProSA [32]. The structural comparison of AmyBa with soybean β-amylase (PDB ID: 1Q6C) and other plant β-amylases (data not shown) revealed the existence of an additional starch-binding domain (SBD) at the microbial β-amylase C-terminus (Fig. 1B). A previous study suggested that the lack of an SBD in plant β-amylases might result in its ability to exist in starch-rich environments [11], which is notably different from microbial β-amylases.

### Heterologous expression and purification of AmyBa

The *AmyBa* DNA fragment was subcloned into the pBE-S plasmid with the aprE promoter and engineered with an aprE signal sequence to direct Sec-dependent secretion [33]. The resulting expression plasmid (*AmyBa*/pBE-S) was then used for protein expression in *B. subtilis* TEB1030. The recombinant strain was incubated at 37 °C, and samples were taken after 12 h, 24 h, 36 h, and 48 h of growth. Notably, at 36 h, we observed the highest extracellular levels of AmyBa (1590.6 U/mL). Recently, strain screening, fermentation optimization, and heterologous recombinant expression have been applied to improve β-amylase production; however, the low fermentation activity of β-amylase remains a problem. Recently, *Paenibacillus chitinolyticus* CKS1 was obtained, and the fermentation conditions were optimized, revealing a maximum β-amylase production of 2.2 U/mL [17]. Additionally, *T. thermosulfurigenes* β-amylase was heterologously expressed in *Escherichia coli*, achieving the highest β-amylase production (215.0 U/mL) [34].

**Fig. 3:**
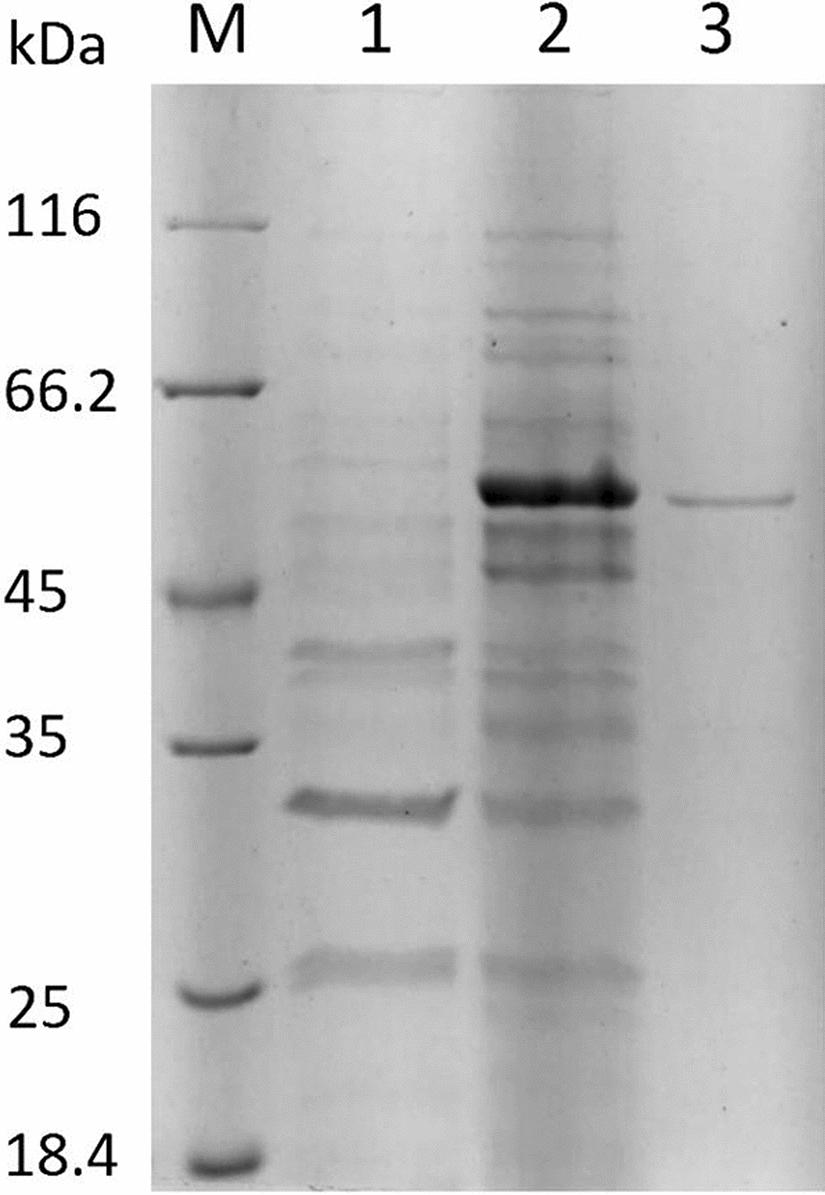
SDS-PAGE analysis of AmyBa. Lanes: M: protein standard; 1: fermentation supernatant of *B. subtilis* TEB1030 (pBE-S); 2: fermentation supernatant of *B. subtilis* TEB1030 (*AmyBa*/pBE-S); and 3: fraction from the Superdex 200 Increase 10/300 GL column

We purified recombinant AmyBa from the cell-free culture supernatant, and following ammonium sulfate precipitation and dialysis, the enzyme was subjected to gel filtration chromatography. Pooled fractions showing β-amylase activity were then concentrated and fractionated by gel filtration. SDS-PAGE analysis showed that the purified protein was homogeneous and exhibited a subunit molecular mass of ~ 56 kDa (Fig. 3, lane 3), which agreed with the theoretical molecular mass for the recombinant enzyme. The purification procedure is summarized in Table 2. The specific activity of the purified enzyme was 3798.9 U/mg, and the purification fold and final yield were 4.7 and 9.4%, respectively.

**Table 2:** Purification scheme of recombinant β-amylase

Purification steps	Total protein (mg)	Total activity (U)	Specific activity (U mg^− 1^)	Purification (-fold)	Yield (%)
Crude Enzyme	113.7	92711.0	815.4	1	100
Ammonium Sulfate Fraction	53.6	69927.8	1304.6	1.6	75.4
Superdex™ 200 Increase 10/300 GL	2.3	8737.5	3798.9	4.7	9.4

Table 3 shows that recombinant AmyBa exhibited the highest specific activity among reported mesothermal microbial β-amylases, with results showing 2.53-, 1.74-, and 1.23-fold higher activity relative to those from *P. polymyxa* [35], *B. cereus* [25], and *B. flexus* [36], respectively. A major impediment to wide industrial application of enzymes is the cost. Consequently, a high specific activity, which can shorten the reaction period and reduce enzyme dosage, is among the most important preconditions for industrial enzymes. In the previous 30 years, numerous microbial β-amylases have been identified [11, 13, 15, 18–20, 29, 35, 37, 38], and most of the coding genes have been successfully cloned, heterologously expressed, and characterized. Structural analysis and the capability for molecular modifications (including site-directed mutagenesis and directed evolution) have been subsequently used to explore the catalytic mechanisms and improve the properties of microbial β-amylases [7, 18, 21, 28, 29]. However, the reported microbial β-amylases do not have high enough special activity to meet the application requirements.

**Table 3:** Comparisons of the biochemical properties of various mesothermal microbial β-amylases

Microorganism	GenBank accession no.	Specific activity (U/mg)	Optimum temperature (°C)	Optimum pH	Host	Plasmid	Reference
*B. aryabhattai*	This study	3798.9	50	6.0	*Bacillus subtilis* TEB1030	pBE-S	This study
*B. cereus*	P36924.2	2182	40	7.0	NR	NR	[25]
*B. flexus*	RIV10038.1	3092	50	7.0–8.0	*E.coli* BL21(DE3)	pET24a(+)	[36, 39]
*P. polymyxa*	3VOC_A	1500	45	7.5	*B. polymyxa* No. 26 − 1(WT)	no	[35]
*B. firmus*	P96513.1	NR	55–60	NR	*E.coli* HB101	pUC18	[38]
*B. circulans*	P06547.1	0.77 μm/mg/min	50	7.0	*E.coli* Rosetta2	pET-21a(þ)	[18]
*Halobacillus* sp. LY9	NR	NR	60	8.0	*Halobacillus* sp. LY9(WT)	no	[19]
*B. megaterium*	WP_013081506.1	NR	50	7.5	*B. megaterium* DSM319	pDAMY1	[40]

NR: not reported; WT: wild type strain

### The effects of temperature and pH on AmyBa

**Fig. 4:**
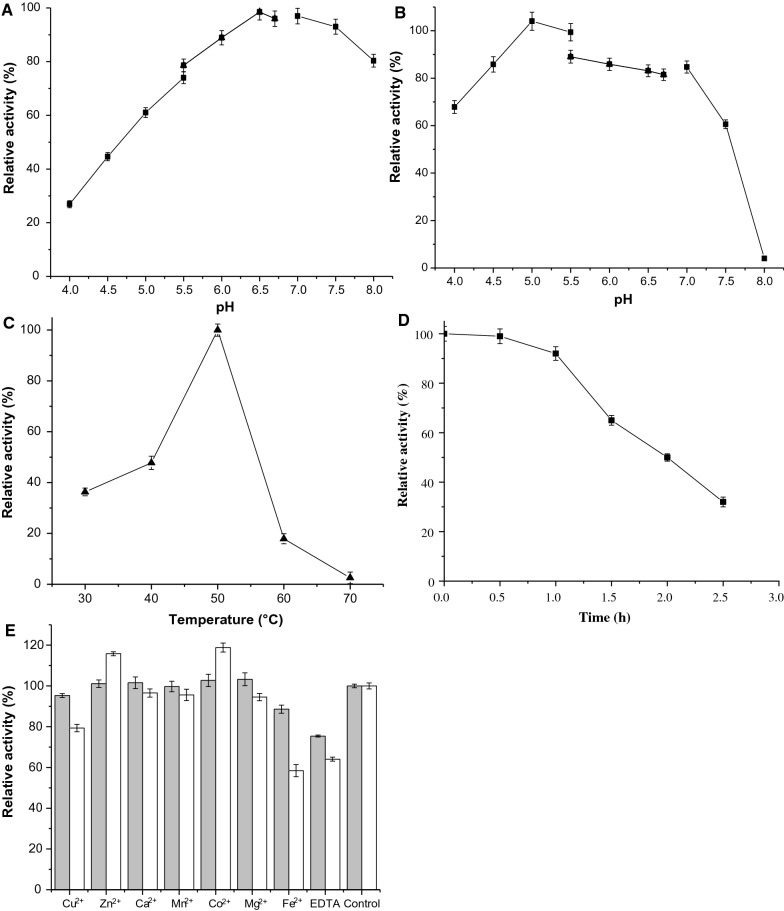
The effects of temperature, pH, and metal ions on AmyBa activity. **A** The effect of pH on AmyBa activity. Reaction were performed in different buffers [50 mM Acetate buffer (4.0–5.5), MES (5.5–6.7), and Tris-HCl (7.0–8.0)] at 50 °C. **B** The effect of pH on AmyBa stability as measured by incubating the enzyme for 12 h in buffer with a pH range of 4.0 to 8.0 at 4 °C. The enzyme activity pre-incubation was established at 100% under optimal conditions. **C** The effect of temperature on AmyBa activity. The reaction was performed in buffer [50 mM MES (pH 6.5)] at different temperatures (30–70 °C) for 10 min. **D** The thermal stability of AmyBa was determined at 50 °C and pH 6.5, with hydrolase activity periodically measured. **E** Effect of metal ions and chelating agents on AmyBa activity (gray, 1 mM; and white, 5 mM)

We found that the enzyme had an optimal pH of 6.5 and retained high activity (> 70%) in a pH range of 5.5 to 8.0 (Fig. 4A). Additionally, recombinant AmyBa maintained a high level of stability under weak acidic to weak alkaline conditions (4.5–7.0) (Fig. 4B), which is consistent with most microbial β-amylases, including those from *B. cereus* [25], *B. flexus* [39], *B. circulans* [18], and *B. megaterium* [40]. However, bacterial and plant β-amylases demonstrate different optimal pH ranges, with a previous reporting maximal activities under neutral and weak acidic conditions, respectively [7].

The optimal temperature for AmyBa activity was measured in a temperature range of 30 to 70 °C, at pH 6.5, revealing an optimal temperature at 50 °C, with 47.8 and 17.9% relative activities at 40 and 60 °C, respectively (Fig. 4C). To evaluate AmyBa thermostability, the enzyme was incubated in sodium phosphate buffer (pH 6.5) at 50 °C, and samples were collected after different incubation times to assess residual activity. The results indicated an enzyme half-life of 2 h at 50 °C (Fig. 4D). Several reports indicate numerous starch hydrolases exhibit an optimal activity at between 50 and 60 °C and under mildly acidic pH conditions [26, 41]. Therefore, we speculate that AmyBa shows great potential for synergistic effects with other amylolytic enzymes, such as α-amylase, pullulanase, and maltogenic amylase.

### The effect of metal ions and EDTA on AmyBa activity

We then pre-incubated purified AmyBa in a reaction mixture containing multiple metal ions (1 mM and 5 mM Cu^2+^, Zn^2+^, Ca^2+^, Mn^2+^, Co^2+^, Mg^2+^, and Fe^2+^) and chelating agents (1 mM and 5 mM disodium EDTA) at 25 °C for 1 h. We found that the presence of chelating agents (1 mM or 5 mM) had a significant inhibitory effect on AmyBa activity (Fig. 4E), suggesting that divalent cations are required for catalysis. Additionally, the presence of 1 mM Mn^2+^ or Zn^2+^, Ca^2+^, Mg^2+^, Co^2+^ resulted in no change in enzyme activity, and Cu^2+^, and Fe^2+^, respectively, resulted in moderate inhibition of activity (retained 88.6–95.3% activity). Furthermore, enzyme activity was inhibited by higher concentration (5 mM) of Cu^2+^ and Fe^2+^ but enhanced by 5 mM Co^2+^ and 5 mM Zn^2+^, respectively (Fig. 4E).

### The kinetic parameters of AmyBa

We then performed kinetic analysis of AmyBa at 50 °C (Table 4), revealing *V*_max_, *k*_cat_, and *K*_m_ values toward soluble starch of 6660.0 ± 577.1 µMol/mg min, 116961.1/s, and 9.9 ± 2.1 mg/mL, respectively. Moreover, the *k*_cat_/*K*_m_ value was 11733.7 mL/s/mg. Previous studies showed that the *k*_cat_, and *K*_m_ values of recombinant *B. flexus* β-amylase was 2805.2 /s, and 85.86 ± 2.1 µM/L, respectively [36], and the recombinant *B. flexus* β-amylase has been commercial produced [39]. Compared with *B. flexus* β-amylase, the AmyBa has higher catalytic rate constants, we speculate that AmyBa shows potential developing value.

**Table 4:** The kinetic paraments of recombinant β-amylase

Kinetic paraments	Data
*V*_max_ (µMol/mg min)	6660.0 ± 577.1
*K*_cat_ (s^− 1^)	116961.1 ± 10134.9
*K*_m_ (mg/mL)	9.9 ± 2.1
*K*_cat_/*K*_m_ (mL/s/mg)	11733.7 ± 1016.7

### Substrate specificity

We then evaluated the relative activity of AmyBa in the presence of a variety of polysaccharide substrates (Table 5). In the presence of soluble starch, AmyBa showed maximal activity (100%), as well as high activity toward cornstarch (> 95%), whereas lower relative activity was observed in the presence of tapioca starch (~ 35%). Additionally, in the presence of a variety of dextrins, AmyBa exhibited higher relative activity for those with a high DE (10–15: 65.2%) and lower for relative activity for those with a lower DE (8–10: 47.8%). These results indicated that recombinant AmyBa showed efficient hydrolysis ability toward soluble starch, cornstarch and dextrin (DE 10–15), which was similar with previous studies evaluating β-amylases from *B. flexus*, *B. polymyxa*, barley, wheat and soybean [35, 39].

**Table 5:** Substrate specificity of recombinant β-amylase

Substrate	Relative activity (%)
Soluble starch	100.0 ± 2.1
Dextrin DE 15–20	69.3 ± 1.7
Dextrin DE 10–15	45.2 ± 1.0
Dextrin DE 8–10	33.1 ± 0.4
Corn starch	95.6 ± 1.3
Tapioca starch	35.3 ± 0.3

### Maltose production from starch catalyzed by recombinant AmyBa

We then applied AmyBa for starch saccharification for maltose production. We first gelatinized a 10% corn starch slurry and liquefied it using high-temperature α-amylase (10.0 U/g starch). We then initiated the saccharification process using pullulanase (1.0 U/g starch) and different β-amylases (15 U/g starch), including recombinant AmyBa, soybean β-amylase, and sweet potato β-amylase. HPLC analysis of samples were taken at regular intervals revealed maximum maltose yields of 55.14%, 51.69%, and 51.19% for recombinant AmyBa, sweet potato β-amylase, and soybean β-amylase, respectively, after 6 h of saccharification. These results indicated that recombinant AmyBa showed efficacy for maltose production. Previous studies evaluating recombinant *B. flexus* β-amylase observed similar results, with the enzyme exhibiting a higher maltose yield (56.3%) than that of barley and wheat β-amylases [39].

### Optimization of conditions for maltose production

**Fig. 5:**
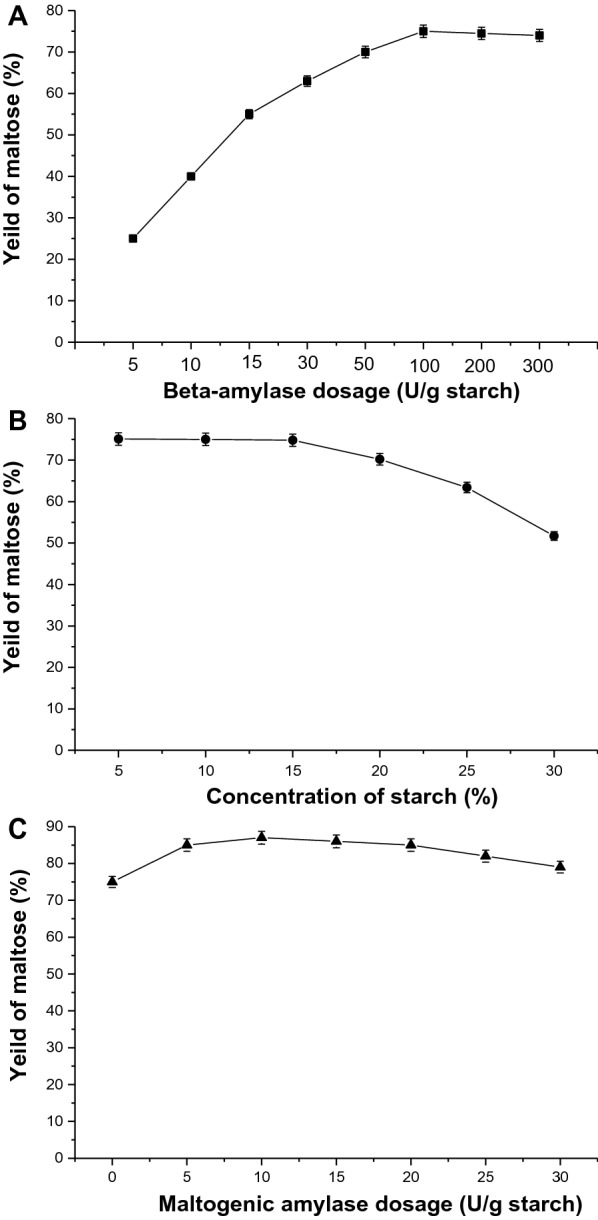
Optimization of maltose production. **A** Effect of AmyBa dosage (black square), **B** starch concentration (black circle), and **C** maltogenic amylase dosage (black triangle) on maltose yield

To further characterize the properties of recombinant AmyBa, we optimized the reaction condition, dosage, starch concentration, and maltogenic amylase dosage. Recent studies report that enzyme dosage plays an important role in enzymatic reaction system [26, 42]. We added different amounts of AmyBa (5, 10, 15, 30, 50 100, 200 and 300 U/g) to a 10% cornstarch solution, followed by sampling and analysis after 6 h. The results showed that the AmyBa dosage significantly impacted maltose yield (Fig. 5A), with maltose content increasing along with increasing AmyBa level (5–100 U/g). The maximum yield of maltose from starch was 75.2%; however, the yield decreased slightly at AmyBa dosages > 100 U/g, indicating that the optimal dosage was 100 U/g starch (Fig. 5A).

We then determined the optimal substrate concentration by preparing various cornstarch solutions (5, 10, 15, 20, 25 and 30%) in sodium phosphate buffer (pH 6.0) with β-amylase and pullulanase concentrations at 100.0 U/g and 1.0 U/g starch, respectively, and subjecting them to reactions at 50 °C for 6 h. At starch concentrations of 5%, 10%, and 15%, maltose yields were 75.1%, 75.0%, and 74.8%, respectively (Fig. 5B), whereas starch concentrations > 15% resulted in decreased maltose yields (20%, 25%, and 30% starch yielded 70.2%, 63.2%, and 51.7% maltose, respectively). This result might be explained by the decreased hydrolytic activity of AmyBa at high substrate concentrations as a consequence of increased interactions between AmyBa and starch or the dextrin chain, which would restrict their movement. Moreover, a higher viscosity in the reaction system might hinder substrate migration and accessibility to the enzyme active site. Furthermore, higher starch concentrations would result in increased ratios of malto-oligosaccharide and isomalto-oligosaccharide products, likely associated with increased by-products of the enzyme reaction.

We found that the hydrolysis products included a large amount of maltotriose (> 15%), with increased byproduct content resulting in difficulties with downstream purification of high maltose syrup. A previous study identified maltogenic amylase and reported its ability to hydrolyze maltotriose to release maltose and glucose [17, 26]. To investigate synergistic effects between AmyBa and maltogenic amylase, we performed two-step saccharification and optimized the maltogenic amylase dosage. After the first step of saccharification, we added different amounts (5, 10, 15, 25, and 30 U/g starch) of maltogenic amylase for the second step, which was performed at 60 °C for 12 h. Reaction samples analyzed using HPLC revealed an increased maltose yield along with increasing amounts of maltogenic amylase up to 10 U/g starch, after which the maltose yield decreased slightly. We identified a maximum yield of maltose (DP2) from starch at 87.0% for the two-step process, and the content of glucose (DP1), maltritose (DP3) and short-chained dextrins (DP4+) was 5.6%, 3.3 and 4.1%, respectively (Fig. 5C). Whereas, the content of glucose (DP1), maltritose (DP3) and short-chained dextrins (DP4+) was 1.52%, 15.0 and 2.25% for the enzyme reaction without maltogenic amylase, respectively (data not shown).

### Docking analysis of AmyBa

**Fig. 6:**
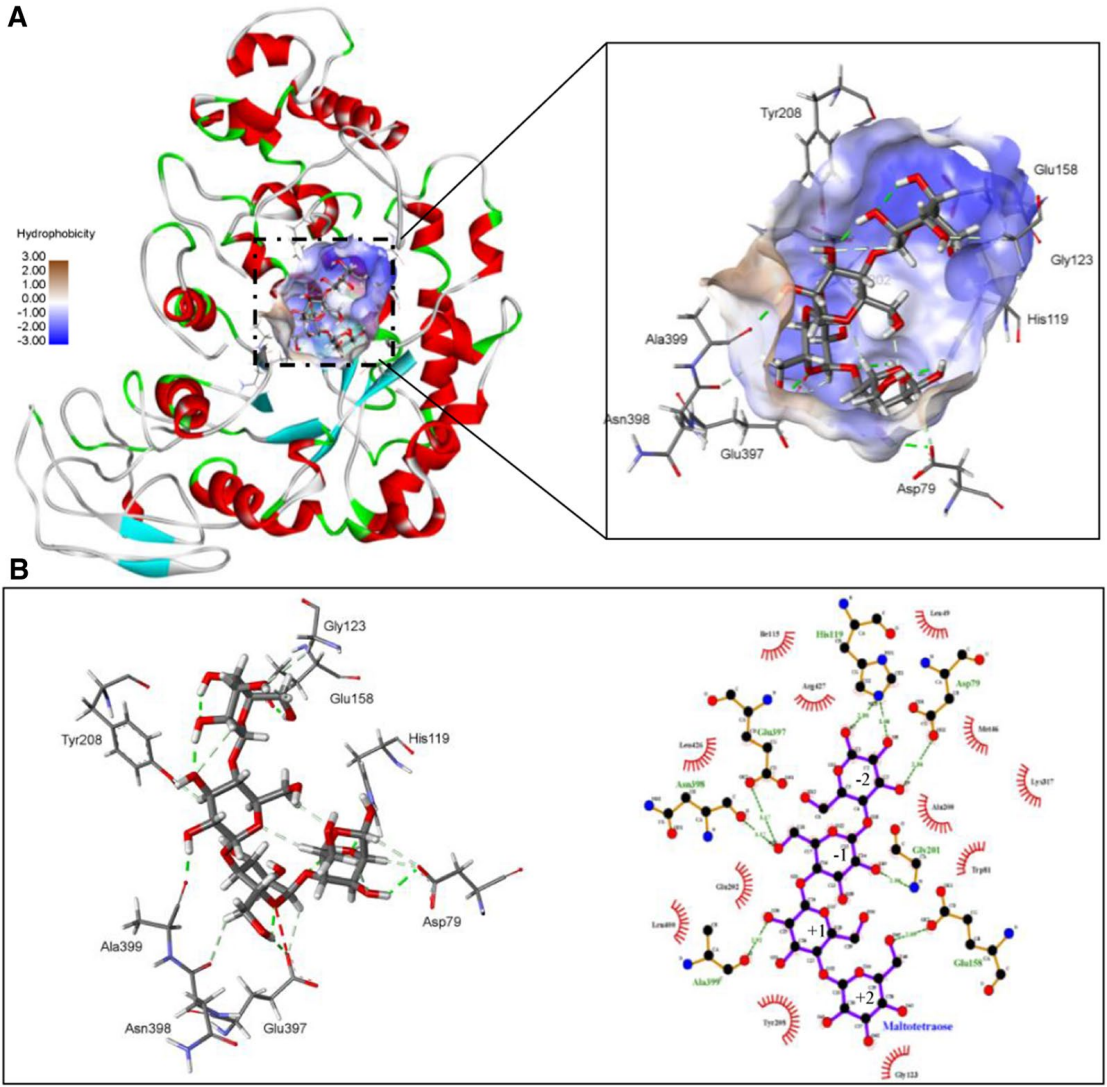
Molecular docking of AmyBa with maltotetraose. **A** Overall structure and substrate binding pocket analysis of AmyBa. **B** Schematic representation showing enzyme (AmyBa) /substrate (maltotetraose) interactions of AmyBa. Hydrogen bonds are shown as green dotted lines

The model AmyBa with least values for DOPE score was selected for docking studies. Maltotetraose was docked with the model to generate binding mode. Molecular docking showed that maltotetraose binds to a substrate binding pocket of the (β/α)_8_-barrel (Fig. 6A).

In order to identify the key amino acid residues responsible for substrate recognition, the enzyme–substrate interactions were analyzed by Yasara, and depicted with Discovery Studio Visualizer 2020 and ligplot. Figure 6B shows the hydrophobic interaction and hydrogen bonding networks at the active pocket. It found that the residues Asp79, His119, Glu159, Gly201, Glu397, Asn398, and Ala399 form hydrogen bonds towards maltotetraose, respectively. At the same time, there are twelve amino acids residues (Met46, Leu49, Trp81, Ile115, Gly123, Ala200, Glu202, Tyr208, Lys317, Leu400, Leu426, and Arg427) forming hydrophobic interaction with maltotetraose. These results indicated that AmyBa has a strong binding ability towards maltotetraose, which is conducive to the binding and hydrolysis of substrate. In addition, it found that there are 6 hydrogen bonds and 6 hydrophobic interaction for glucose residues at subsites − 1 and − 2, however, the hydrogen bonds and hydrophobic interaction was only 2 and 3 for glucose residues at subsites + 1 and + 2, respectively (Fig. 6B and Additional file 1). This suggested that the glucose residues at subsites − 1 and − 2 interacted tightly with the active center of enzyme than that at subsites + 1 and + 2. Furthermore, based on obtained docking pose, the interaction between catalytic residues (Glu202 and Glu397) and substrate was also analyzed. It revealed that the carboxyl group of Glu202 and carboxyl group of Glu397 located on the hydrophilic surface and hydrophobic face of the glucose residue (subsite − 1), respectively. Previously study revealed that the amino acids residues Glu186 and Glu380 of soybean β-amylase play critical roles as a general acid and a general base catalyst, respectively [43]. In this study, based on molecular docking (Fig. 6) and sequence alignment (Fig. 1A), it predicted that the residues Glu202 of AmyBa acts as a proton donor, and the Glu397 of AmyBa acts as catalytic base, which probably involved in activating the attacking water molecule.

As shown in Table 3, the AmyBa exhibited the highest specific activity (3798.9 U/mg) among reported mesothermal microbial β-amylases. It is well known that the catalytic power of enzyme is mainly derived from the binding energy, which is the free energy released in forming the multiple interactions between enzyme and substrate. The interactions can lower activation energies by the 60 to 80 kJ/mol, which resulted in the large enhancements of catalytic rate for enzymes [44]. To reveal the molecular mechanisms of the high catalytic activity for AmyBa, the binding energy of AmyBa to maltotetraose was calculated by Yasara. Meanwhile, the binding energy of three typical β-amylases (*B. cereus*, *P. polymyxa* and *Sweet Potato*) to maltoteraose was also calculated and compared. It showed that the binding energy for AmyBa, *B. cereus* β-amylase, *P. polymyxa* β-amylase and *Sweet Potato* β-amylase was − 113.82, -84.37, -78.51, and − 144.36 kJ/mol. It also found that the specific catalytic activity of AmyBa, *B. cereus* β-amylase, *P. polymyxa* β-amylase and *Sweet Potato* β-amylase was 3798.9, 2182.3, 1500.0, and 3897.2 U/mg, respectively. Comparison of the binding energy and specific activity between different β-amylases suggested that experimental catalytic power of different β-amylases is in good agreement with its substrate binding energy. This observation is consistent with reported in the literature, that binding energy of the enzyme–substrate complex is the dominant driving force to catalysis [45].

## Conclusions

In summary, we cloned a β-amylase-encoding (*AmyBa*) from *B. aryabhattai* and performed heterologous expression to obtain the recombinant enzyme, which was characterized in detail. Multiple sequence alignment showed that AmyBa shares > 45% identity with other microbial β-amylases but lower identity with plant β-amylases. Additionally, optimization of the reaction conditions for the production of maltose from starch and use of two-step saccharification resulted in a maximal yield of 87% from conversion of 10% starch by AmyBa and maltogenic amylase. Notably, AmyBa exhibited the highest specific activity among reported mesothermal microbial β-amylases, suggesting its status as a promising candidate for use in the industrial production of maltose from starch.

## Materials and methods

### Strains and vectors

*Bacillus* sp. Gel09, a wild-type β-amylase-producing strain, was previously isolated from soil and identified as *B. aryabhattai* CCTCC M2017320 in our laboratory (Food Enzyme Lab). *Escherichia coli* JM109 and *Bacillus subtilis* TEB1030 were used as hosts for gene cloning and expression, respectively, and pMD18-T and pBE-S (Takara Biotechnology Co, Ltd., Beijing, China) were used as cloning and expression vectors, respectively.

### Enzymes and chemicals

Restriction enzymes (*Nde*I and* Hind*III), DNA polymerase PrimerSTAR HS and rtaq, calf intestine alkaline phosphatase, agarose, and nucleic acid electrophoresis standards were purchase from Takara Biotechnology Co, Ltd. The bacterial genomic DNA extraction kit, agarose gel DNA recovery kit, and EZ-10 spin column plasmid mini-prep kit were obtained from Tiangen Biotech Co., Ltd (Beijing, China). PCR primers were synthesized by Genscript Biotech Corporation (Nanjing, China). The protein electrophoresis standards and polyacrylamide gel electrophoresis kit were obtained from Beyotime Biotechnology (Shanghai, China). α-Amylase, pullulanase, and maltogenic amylase were obtained from Novozymes (Copenhagen, Denmark). All other chemicals were purchased from Sinopharm Chemical Reagent Co., Ltd. (Shanghai, China) unless otherwise indicated.

### DNA manipulation

The genomic DNA of *B. aryabhattai* CCTCC M2017320 was extracted and purified using the Tiangen bacterial genomic DNA extraction kit according to manufacturer instructions. Based on information for *B. aryabhattai* β-amylases in GeneBank, we designed two primers (amy-F: 5′-CCGGCGATGGCATATGGTAGATGGAAAATCAATGAATCC-3′ and amy-R: 5′- GTGCGGCCGCAAGCTTACCAATTATCTGTATAAGTTGC-3′) for amplification (underlined areas are* Nde*I and* Hind*III restriction sites). The gene encoding β-amylase (*AmyBa*) was amplified by PCR using *B. aryabhattai* CCTCC M2017320 genomic DNA as template and the following PCR conditions: 94 °C for 4 min, followed by 30 cycles of denaturation at 94 °C for 30 s, annealing at 57 °C for 30 s, and extension at 72 °C for 1 min 30 s, with a final extension at 72 °C for 20 min. The PCR product was purified and ligated into the pMD18-T vector and transformed into *E. coli* JM109.

The resulting plasmids were verified by sequencing and then digested with *Nde *I and *Hind* III. After electrophoresis and gel-band purification, the DNA fragment encoding β-amylase was ligated into the *Nde *I- and *Hin*d III-digested pBE-S vector. The ligation mixture was then used to transform *E. coli* JM109 cells, followed by confirmation of the recombinant plasmid (pBE-S-AmyBa) by restriction enzyme analysis and DNA sequencing. The verified plasmid was then used to transform *B. subtilis* TEB1030 for expression.

### Sequence analysis

The nucleotide sequences and predicted amino acid (aa) sequences were analyzed using DNAMAN (v.9.0; Lynnon Biosoft, Ramon, CA, USA). The NCBI ORFfinder tool was used to predict the open reading frame. Multiple sequence alignment of AmyBa with other β-amylases was performed using Clustal Omega (clustal.org/omega/) and rendered with ESPript (v.3.0; http://espript.ibcp.fr/ESPript/ESPript/). The phylogenetic tree was constructed using the neighbor-joining method with MEGA software (v.7.0; https://www.megasoftware.net/) to analyze the evolutionary relationships between different sources of β-amylase. The signal peptide was predicted using SignalP (v.5.0; http://www.cbs.dtu.dk/services/SignalP/), and ExPASy (Compute pI/Mw; https://www.expasy.org/) was used to predict the molecular weight and isoelectric point (pI) of the enzyme.

### Expression and purification of recombinant β-amylase in *B. subtilis*

To express recombinant β-amylase, transformed *B. subtilis* TEB1030 single-colony cells were inoculated into LB broth containing kanamycin (10 µg/mL) and grown for 8 to 10 h at 37 °C with shaking at 200 rpm. The culture was then inoculated into TB medium and shaken at 200 rpm for 48 h at 37 °C. The supernatant was collected as the crude-enzyme fraction after centrifugation at 8000 rpm for 10 min, and recombinant enzyme was purified using ammonium sulfate precipitation and dialysis, then the enzyme was subjected to gel filtration chromatography (SuperdexTM 200 Increase 10/300 GL; GE Healthcare, Pittsburgh, PA, USA). Fractions exhibiting β-amylase activity were pooled, and assayed for purity and subunit molecular weight by 12% sodium dodecyl sulfate polyacrylamide gel electrophoresis (SDS-PAGE). 20–200 µg protein was used for SDS-PAGE assay. Protein concentration was measured by the method of Bradford using bovine serum albumin as the standard.

### Enzyme assay

β-amylase activity was determined in 50 mM MES (pH 6.5) using soluble starch as a substrate according to methods described previously [19], with slight modification. Briefly, 0.5 mL of appropriately diluted enzyme solution was added to 0.5 mL 2% (w/v) of soluble starch in 50 mM MES (pH 6.5) and incubated at 50 °C for 10 min. We then added 0.8 mL of 3,5-dinitrosalicylic acid solution and incubated the mixture in a boiling water bath for 5 min, after which 11.2 mL of deionized water was added to dilute the mixture, and absorbance was determined at 540 nm. Maltose was used to generate the standard curve. One unit of β-amylase activity was defined as the rate of enzyme required to release 1 µM of reducing sugars per min under the assay conditions specified.

### Characterization of AmyBa

The optimal pH for β-amylase was examined over a pH range of 4.0 to 7.0 using different buffers, including sodium acetate buffer (pH 4.0-5.5), MES (pH 5.5–6.7), and Tris-HCl (pH 7.0–8.0). The optimal temperature for β-amylase was determined in 50 mM MES buffer (pH 6.5) in a temperature range of 30 to 70 °C. 0.5–3.0 U AmyBa was used in the activity assay.

The pH stability of the enzyme was determined by incubation in different buffers with pH values ranging from 4.0 to 8.0 [using different buffers, including sodium acetate buffer (pH 4.0-5.5), MES (pH 5.5–6.7), and Tris-HCl (pH 7.0–8.0)] at 4 °C overnight. Residual activities were measured under standard conditions, and the thermostability of the enzyme was determined as half-life during treatment at 50 °C. 0.5–3.0 U AmyBa was used in the activity assay. The initial activity before treatment at 50 °C was established as 100%.

AmyBa was pre-incubated with different metal ions (1 mM and 5 mM Cu^2+^, Zn^2+^, Fe^2+^, Ca^2+^, Mn^2+^, Co^2+^, and Mg^2+^) and chelating agents (1 mM and 5 mM disodium EDTA) at 25 °C in 50 mM MES (pH 6.5) for 1 h, and the residual activities were measured under standard assay conditions. 0.5-3.0 U AmyBa was used in the activity assay.

### Substrate specificity and kinetic parameter determination

The ability of purified recombinant β-amylase to hydrolyze various substrates was examined at 50 °C in 50 mM MES (pH 6.5). The substrates tested included soluble starch; dextrin with dextrose equivalent (DEs) of 15–20, 10–15, and 8–10; corn starch; and tapioca starch at a concentration of 1% (w/v).

The kinetic parameters of the enzyme were determined using soluble starch as a substrate at 12 different concentrations (0.5, 1.0, 2.0, 3.0, 5.0, 7.0, 9.0, 10.0, 15.0, 20.0, 30.0, and 40.0 mg/mL). The enzymatic reactions were performed in 50 mM MES (pH 6.5) at 50 °C for 10 min. The *K*_m_, *V*_max_, and *k*_cat_ values were calculated using Lineweaver–Burk plots and the Michaelis–Menten equation using Graphpad Prism software (GraphPad Software, San Diego, CA, USA). 0.5–3.0 U AmyBa was used in the activity assay.

### Maltose production from starch catalyzed by recombinant AmyBa

The application effect of the recombinant AmyBa was analyzed as follows. First, 10% (m/v) cornstarch suspended in 50 mM MES (pH 6.5)) was gelatinized at 95 °C with stirring for 30 min, followed by the addition of thermostable α-amylase (capable of processing 10 U/g starch; Novozymes) and incubation at 95 °C for 30 min to liquefy the starch slurry. The pH was then adjusted to 4.0, and the temperature increased to 100 °C to inactivate the enzyme, followed by cooling to 50 °C and pH adjustment to 6.0. β-amylase (15 U/g starch) and pullulanase (1 U/g starch) were then used for one-step saccharification with incubation in a shaking water bath (200 rpm) at 50 °C for 6 h. Samples were then removed and pretreated at 100 °C for 10 min to inactivate the enzymes.

### Optimization of the reaction conditions for maltose production

To further characterize the saccharification performance of AmyBa, we optimized the enzyme dosage, starch concentration, and maltogenic amylase dosage, respectively.

To investigate the effect of the β-amylase dosage on maltose production, different amounts of recombinant enzyme (5, 10, 15, 30, 50 100, 200, and 300 U/g starch) were used during the one-step saccharification process under conditions described in Sec. 2.9).

The effects of starch concentration were investigated using different cornstarch solutions (5, 10, 15, 20, 25 and 30% cornstarch) at β-amylase and pullulanase concentrations of 100.0 U/g and 1.0 U/g starch, respectively, and at 50 °C. After 6 h of saccharification, samples were analyzed.

To further improve the maltose yield, we employed two-step saccharification and optimized the maltogenic amylase dosage. After one-step saccharification, different amounts (5, 10, 15, 25, and 30 U/g starch) of maltogenic amylase was added to further hydrolyze the malt-oligosaccharide (two-step saccharification), and the reaction was allowed to proceed for an additional 12 h at 60 °C. Reaction products were sampled and analyzed using high-performance liquid chromatography (HPLC).

### HPLC analysis

After cooling, the samples were diluted (1:10, v/v) using deionized water and then filtered (0.45 μm). HPLC analysis was performed using an Agilent 1260 HPLC system (Agilent Technologies, Santa Clara, CA, USA) and a NH2P-504E column (4.6 × 250 mm; Shodex, Tokyo, Japan) at 40 °C, with a mobile phase of 75% (v/v) acetonitrile and a flow rate of 1.0 mL/min. Analysis was performed with a refractive index detector [26].

### Homology modeling and docking analysis

Homology modeling was performed using the Modeller 9.25 package (https://salilab.org/modeller/) and the structure of *Bacillus cereus* β-amylase [Protein Data Bank (PDB) ID: 1J10] as a template. 30 models were generated for AmyBa, the model with the lowest discrete optimized protein energy (DOPE) score was chosen for further analysis. The ligand (maltotetraose) was drawn by ChemDraw 18.0. Then the ligand and receptor were prepared, followed by docking using standard the ligand docking protocol with the Yasara software (Yasara Biosciences GmbH, Vienna, Austria). The model and docking solutions were visualized and analyzed using Yasara software, BIOVIA Discovery Studio Visualizer (DSV) 2020 and ligplot.

## Supplementary Information

**Additional file 1.** Molecular docking related data.

## Data Availability

All data generated or analysed during this study are included in this published article.

## Abbreviations

AmyBa: *Bacillus aryabhattai* β-amylase
CBM: Carbohydrate-binding domain
CCTCC: China Center for Type Culture Collection
DE: Dextrose equivalent
DEAE: Diethylaminoethyl
DOPE: Discrete optimized protein energy
DP: Degree of polymerization
EDTA: Ethylenediaminetetraacetic acid
HPLC: High-performance liquid chromatography
LB: Luria-Bertani
Mw: Molecular weight
PCR: Polymerase chain reaction
PDB: Protein Data Bank
pI: Isoelectric point
SBD: Starch-binding domain
SDS-PAGE: Sodium dodecyl sulfate-polyacrylamide gel electrophoresis
*K*_cat_: Turnover numbers
*K*_m_: Michaelis Constant
*V*_max_: Maximal Velocity
